# Effect of dorsal capsular imbrication on intraoperative DRUJ instability following arthroscopic TFCC repair surgery

**DOI:** 10.1186/s12891-024-07663-z

**Published:** 2024-07-15

**Authors:** Chen-Wei Yeh, Cheng-En Hsu, Tsung-Yu Ho, Wei-Chih Wang, Alvin Kai-Xing Lee, Bor-han Wei, Yung-Cheng Chiu

**Affiliations:** 1 School of Medicine, China Medical University, Taichung, 404 Taiwan; 2 Department of Orthopedic Surgery, China Medical University Hospital, No. 2, Yude Rd., North Dist, Taichung, 404 Taiwan; 3 Sports Recreation and Health Management Degree Program, Tunghai University, Taichung, 407 Taiwan; 4 Department of Orthopedics, Taichung Veterans General Hospital, Taichung, 407 Taiwan; 5 Department of Orthopedic Surgery, China Medical University Hsinchu Hospital, Hsinchu, 302 Taiwan; 6 Cheng Ching Hospital Chung Kang Branch, Taichung, 407 Taiwan

**Keywords:** Foveal repair, Capsular repair, DRUJ instability, DRUJ capsule imbrication, TFCC tear, Level of evidence: level III

## Abstract

**Background:**

To assess the clinical outcomes and identify the ideal indication for implementing dorsal distal radioulnar joint (DRUJ) capsular imbrication after triangular fibrocartilage complex (TFCC) repair in cases of DRUJ instability.

**Methods:**

We conducted a retrospective study on patients who underwent arthroscopic TFCC repair between 2016 and 2021. Inclusion criteria comprised a symptomatic ulna fovea sign for over 6 months and dorsal DRUJ subluxation on magnetic resonance imaging. A total of 225 patients were divided into two groups: Group 1 (135 cases) with a negative ballottement test after “Cross-form TFCC repair” (CR) and Group 2 (90 cases) with a positive ballottement test after “Cross-form TFCC repair” and augmented DRUJ stability through dorsal DRUJ capsular imbrication (CR + DCI). Pain visual analog scale score (VAS), grip strength, modified Mayo Wrist Score (MMWS), wrist range of motion (ROM), and patient-reported outcomes (PROMs) were assessed for a minimum of 3 years postoperatively.

**Results:**

Both groups showed significant improvements in pain VAS score, grip strength, wrist ROM, MMWS, and PROMs between the preoperative and postoperative periods (all *P* < 0.05). Recurrent DRUJ instability occurred in 3.7% and 1.1% of patients in the “CR” and “CR + DCI” groups, respectively, with a significant difference. Despite the “CR + DCI” group initially exhibiting inferior ROM compared with the “CR” group, subsequently, no significant difference was noted between them.

**Conclusions:**

Dorsal DRUJ capsular imbrication effectively reduces postoperative DRUJ instability rates, enhances grip strength, and maintains wrist ROM in patients with a positive intra-operative ballottement test after arthroscopic TFCC repair.

## Introduction

Distal radioulnar joint (DRUJ) stability during unrestricted forearm rotation relies on several factors, including the integrity of the triangular fibrocartilage complex (TFCC), the interosseous membrane, the bony configuration of the sigmoid notch, DRUJ capsule, and the extensor carpi ulnaris tendon with its subsheath [1]. TFCC injury often results from a fall on the outstretched, pronated, and hyperextended wrist, leading to dorsal instability of the DRUJ [2].

Within the anatomical structure of the TFCC, the fovea ulnaris serves as the convergent point of proximal component TFCC (pc-TFCC) insertion, thereby becoming the most indispensable stabilizer for the ulnocarpal joint and DRUJ [3]. Based on the ulnar-side TFCC tear in Palmar type Ib, Atzei et al. [4]. classified the treatment-oriented TFCC peripheral tear into five subgroups depending on whether the distal component (dc-TFCC) or the pc-TFCC was involved. Specifically, Atzei class II and III indicate DRUJ instability with complete and pc-TFCC rupture, respectively [5]. Consequently, the current approach for foveal-involved TFCC tear aims at achieving anatomical TFCC foveal reattachment, which can be accomplished through transosseous sutures [6–8] or suture anchor fixation [4, 9, 10] and has shown satisfactory outcomes.

A prior study revealed that even when radiography findings are negative in patients experiencing post-traumatic wrist pain, 42% of them receive a diagnosis of TFCC injuries [3]. and neglecting severe TFCC tears often leads to chronic DRUJ instability. According to tissue-engineering theory, the interface of bone-to-ligament may not regenerate after injury, resulting in a high rupture recurrence rate [11], and direct bone-to-ligament repair in the chronic stage might exhibit decreased healing potential with the disadvantageous repair margin [12]. Compared with transosseous sutures, transcapsular repair, involving ligament-to-capsular healing, is an alternative method for addressing TFCC fovea tear [13]. In particular, research has supported the notion that transcapsular repair alone can stabilize the DRUJ while achieving anatomical restoration of the dorsal subluxation of the ulna head [13, 14].

However, the integrity of DRUJ surrounding tissues, such as dorsal and volar radioulnar ligaments (DRUL and VRUL) with a superficial and a deep portion attached to the dorsal capsule, needs to be considered after the completion of TFCC repair [15]. For instance, Liu et al. [16]. reported post-operative DRUJ instability rates of 12.1% with capsular repair and 10.1% with fovea transosseous repair. Consequently, additional procedures to reinforce DRUJ stability may be necessary.

The dorsal capsular imbrication (DCI) technique has been proposed and reported to yield positive clinical results in chronic DRUJ dislocation cases [17–25]. However, the use of DCI as a reinforcement procedure in TFCC repair operations for chronic DRUJ instability has not been extensively studied. Therefore, this study aimed to evaluate the surgical outcomes of DCI in patients with a positive intra-operative ballottement test (grade I, II, III) after completing TFCC repair. We hypothesize that this procedure is a safe and reliable treatment option for refractory DRUJ instability in arthroscopic TFCC repair operations.

## Methods

### Patient enrolment

This study adhered to the tenets of the Helsinki Declaration and was approved by the Research Ethics Committee of China Medical University Hospital, Taichung, Taiwan (IRB number: CMUH112-REC2-144). We retrospectively reviewed patients with repairable TFCC (Atzei II, III) injuries who underwent arthroscopic “cross-form” trancapsular repair with or without dorsal DRUJ capsular imbrication from January 2016 to January 2021. A minimum follow-up period of 36 months was mandatory for inclusion. The exclusion criteria encompassed patients with non-repairable TFCC (Atzei IV) ulnar and DRUJ osteoarthritis changes (Atzei V). All procedures were performed by a senior hand surgeon.

### Clinical and image assessment

Pre-operatively, patients were diagnosed via a series of physical examinations, including ulna fovea sign, push-off test, and ballottement test for DRUJ laxity [18]. Wrist X-rays were employed to assess bony structure malalignment, such as ulnar styloid fracture, ulna variance, distal radius fracture or Galeazzi fracture [26]. Additionally, magnetic resonance imaging (MRI) of the wrist was performed to evaluate the condition of articular cartilage wear, detect foveal TFCC tear, and identify ulna head subluxation [27].

### Arthroscopic assessment

Radiocarpal joint arthroscopy was performed using a 3/4 viewing portal (2.7-mm arthroscopy), a 6R working portal (equipped with a synovial shaver and probe), and a 6U portal (utilized as a fluid outflow portal). The 3/4 viewing portal allows the visualization of the dc-TFCC lesion over the ulnar margin of the TFCC. Through the 6R portal, a probe was used to perform a hook test, and a shaver served as a suction test to evaluate the pc-TFCC condition. Notably, in cases where the pc-TFCC was challenging to assess using the aforementioned tests, especially if dc-TFCC was intact (Atzei III), a direct foveal (DF) portal was established. **Then**,** the interposed fibrous and scar tissue were debrided completely with the shaver**,** burr and radiofrequency device. The tourniquet was deflated to check the peripheral vascularization of the TFCC foveal region; after confirming adequate debridement**,** the tourniquet was inflated again.**

### Arthroscopy-assisted “cross-form” tfcc capsular repair with/without dorsal druj capsule imbrication

The detailed procedure for TFCC repair was described as follows:

### Part 1: “Cross-form” TFCC transcapsular repair

Under the aforementioned 3/4 placement with an inflated tourniquet, a combination of 2 − 0 ETHIBOND (Johnson & Johnson, Hamburg, Germany) and 2 − 0 prolene (Ethicon Inc., Somerville, NJ, USA) were combined using the inside-out [28] and outside-in [29] TFCC capsular repair techniques, and a 21-gauge spinal needle was employed to perform the two horizontal stitches.

**Fig. 1:**
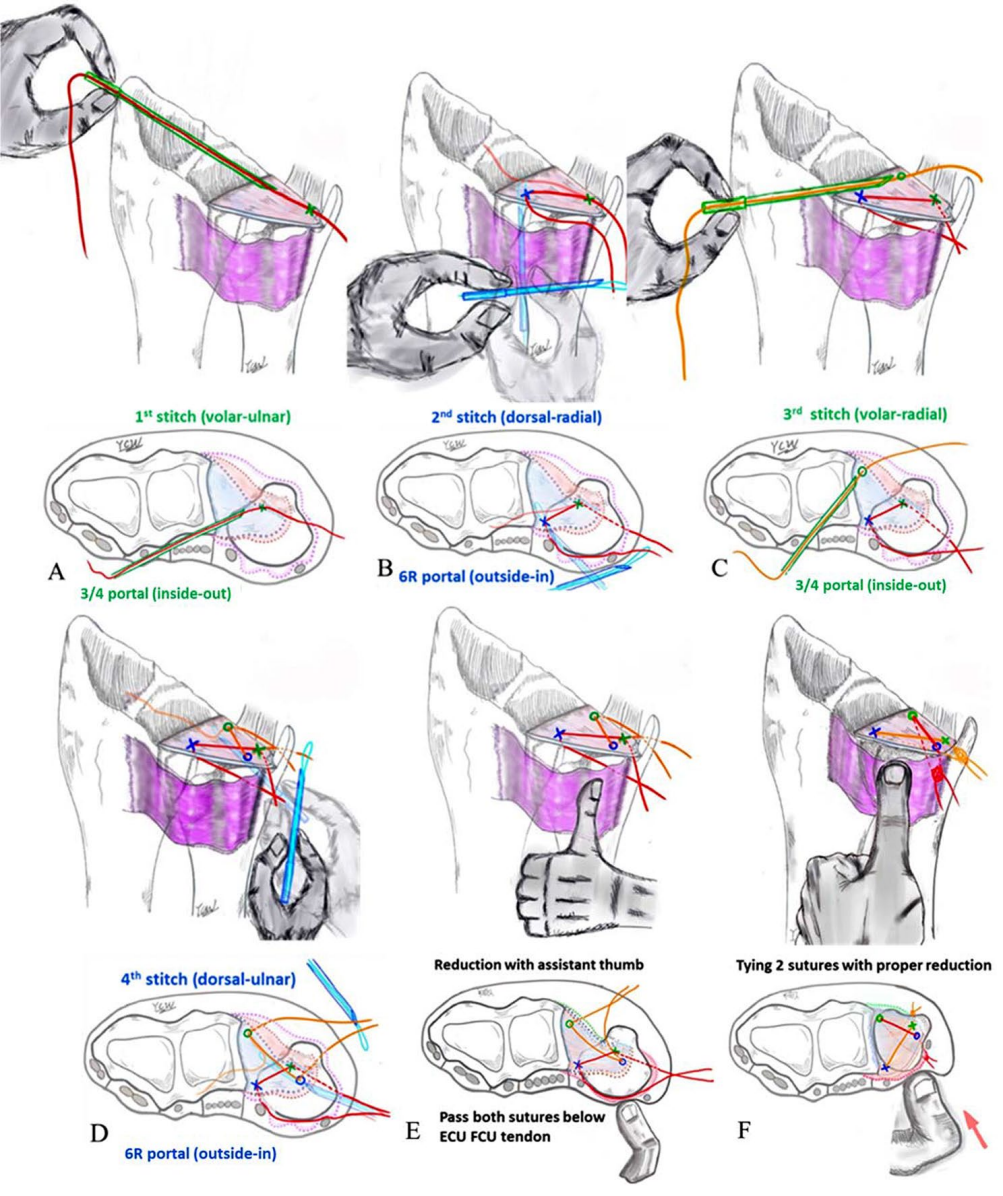
Part 1 ?Cross-form? TFCC transcapsular repair. **(A)** 1st stitch [volar-ulnar]:inside-out technique from the 3/4 portal **(B)** 2nd stitch [dorsal-radial]: outside-in technique from the 6R portal; **(C)** 3rd stitch [volar-radial]: inside-out technique from 3/4 portal **(D)** 4th stitch [dorsal-ulnar]: outside-in technique from 6R portal. **(E)** and **F**) reduction of the ulna head into the radius sigmoid notch with the assistant?s thumb and tying both sutures. Legends: Green color: 3/4 portal, inside-out techniqueBlue color: 6R portal, outside-in technique. Red color: First suture. Orange color: Second suture

**Fig. 2:**
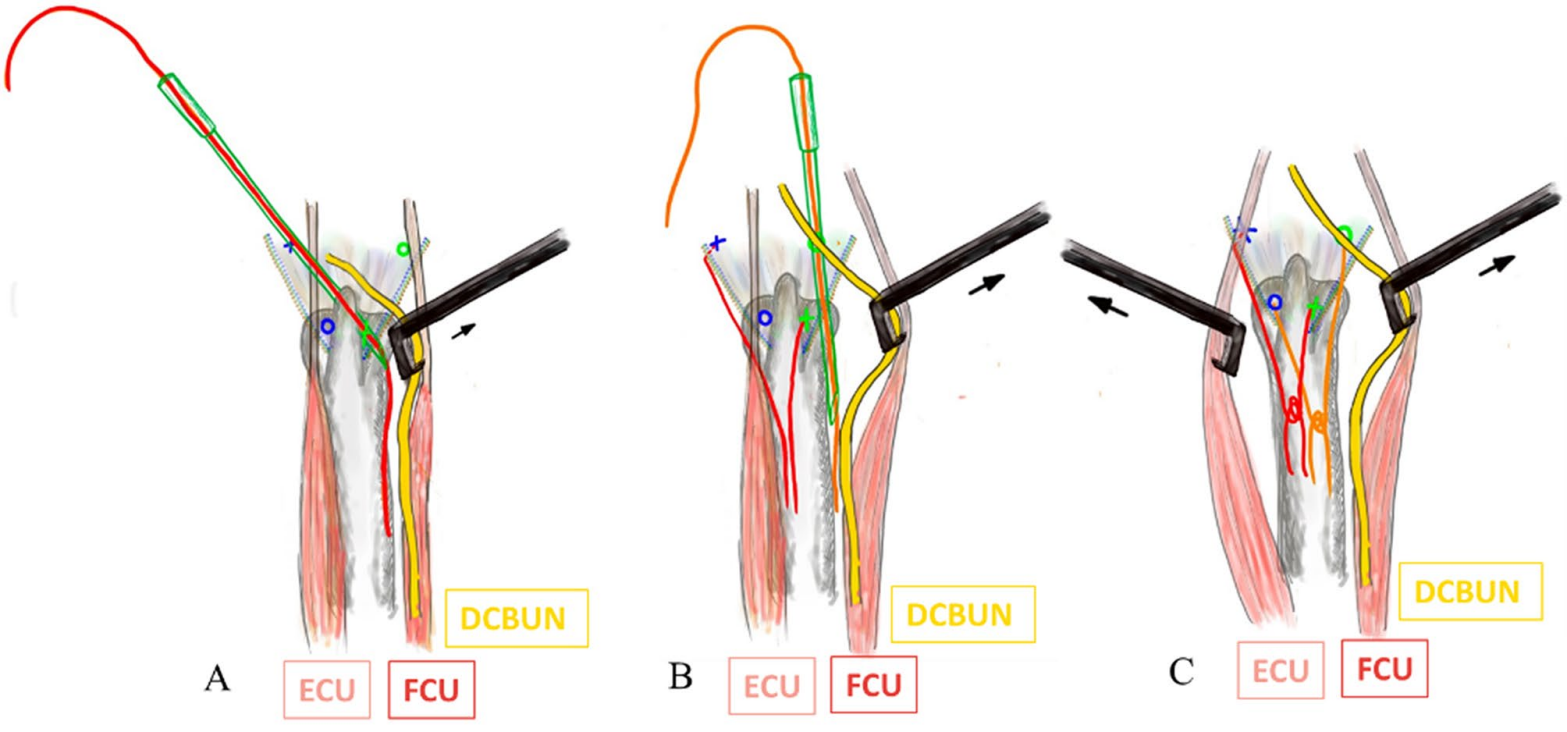
Identify DCBUN and FCU. ( **A**) Applied the first stitch after retracting DCBUN & FCU. (**B**) Applied the third stitch after retracting DCBUN & FCU. ( **C**) Retracting the ECU and DCBUN and FCU, and tying both sutures below them. Legends: Green color: 3/4 portal, inside-out technique. Blue color: 6R portal, outside-in technique. Red color: 1st suture. Orange color: 2nd suture. DCBUN: dorsal cutaneous branch ulna nerve. ECU: Extensor carpi ulnaris. FCU: Flexor carpi ulnaris

**Fig. 3:**
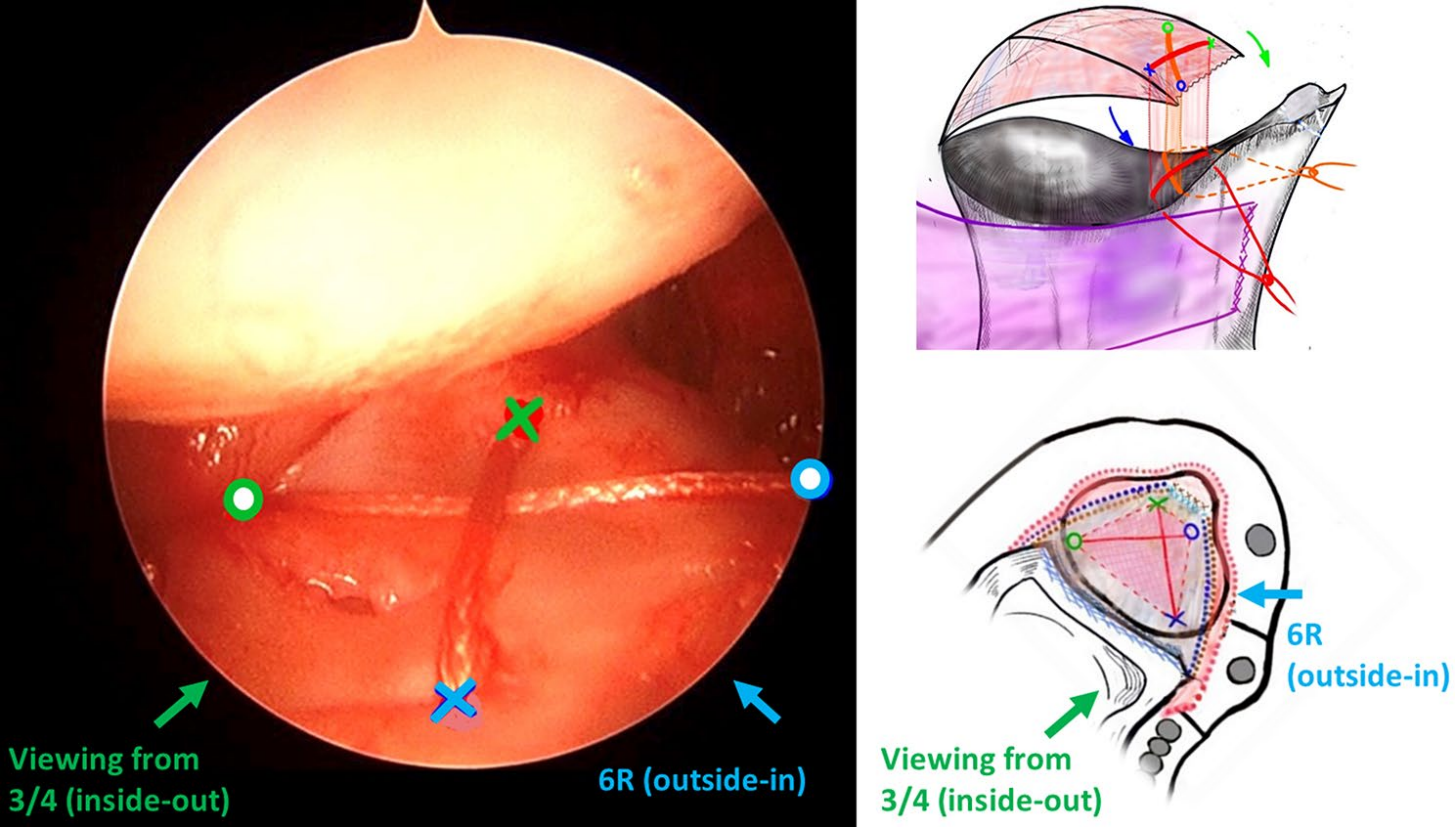
Creating a maximum area of “Cross-form” TFCC transcapsular repair under arthroscopy (Viewing from 3/4 portal). Green color: 3/4 portal, inside-out technique. Legends: Blue color: 6R portal, outside-in technique. Red color: 1st suture. Orange color: 2nd suture. TFCC: triangular fibrocartilage complex

Before suturing, a 2-cm incision was made over the 6U portal. The dorsal cutaneous branch of the ulnar nerve (DCBUN) and flexor carpi ulnaris (FCU) tendon were identified and retracted. The first horizontal mattress suture involved a 2 − 0 ethibond stitch close to the volar-ulnar margin of the TFCC lesion through the 3/4 portal using an inside-out technique (Fig. 1A) and subsequently retracted to avoid DCBUN and FCU involvement (Fig. 2A). The second stitch, a 2 − 0 prolene lasso loop suture, was performed near the dorsal-radial margin of the intact TFCC part through the 6R portal using an outside-in technique. The lasso loop suture carried one end of the 2 − 0 ethibond to form the first horizontal mattress suture (Fig. 1B). For the second horizontal mattress suture, the puncture site of the third stitch was performed close to the volar-radial margin of the TFCC intact part through the 3/4 portal with an inside-out technique (Fig. 1C), and the DCBUN should be protected from being punctured or tied in this step (Fig. 2B). The fourth stitch, a lasso suture, was performed near the dorsal-ulnar margin of the TFCC lesion through the 6R portal using an outside-in technique. The lasso loop suture was then used to carry one end of the third stitch to form the second horizontal mattress suture (Fig. 1D). This “cross-form” TFCC capsular repair created an extensive contact area in the ligament to capsule suture (Fig. 3).

After completing two horizontal mattress sutures, the wrist traction tower device was released and firmly tied in the wrist’s full-pronation position (Fig. 1E). Both sutures were checked to ensure they were tied below the ECU, FCU and DCBUN to avoid neuro-tendon involvement (Fig. 2C), achieved by reducing the ulnar head from dorsal subluxation into a neutral position using thumb compression by an assistant (Fig. 1F).

### Part 2: intra-operative ballottement test

**Fig. 4:**
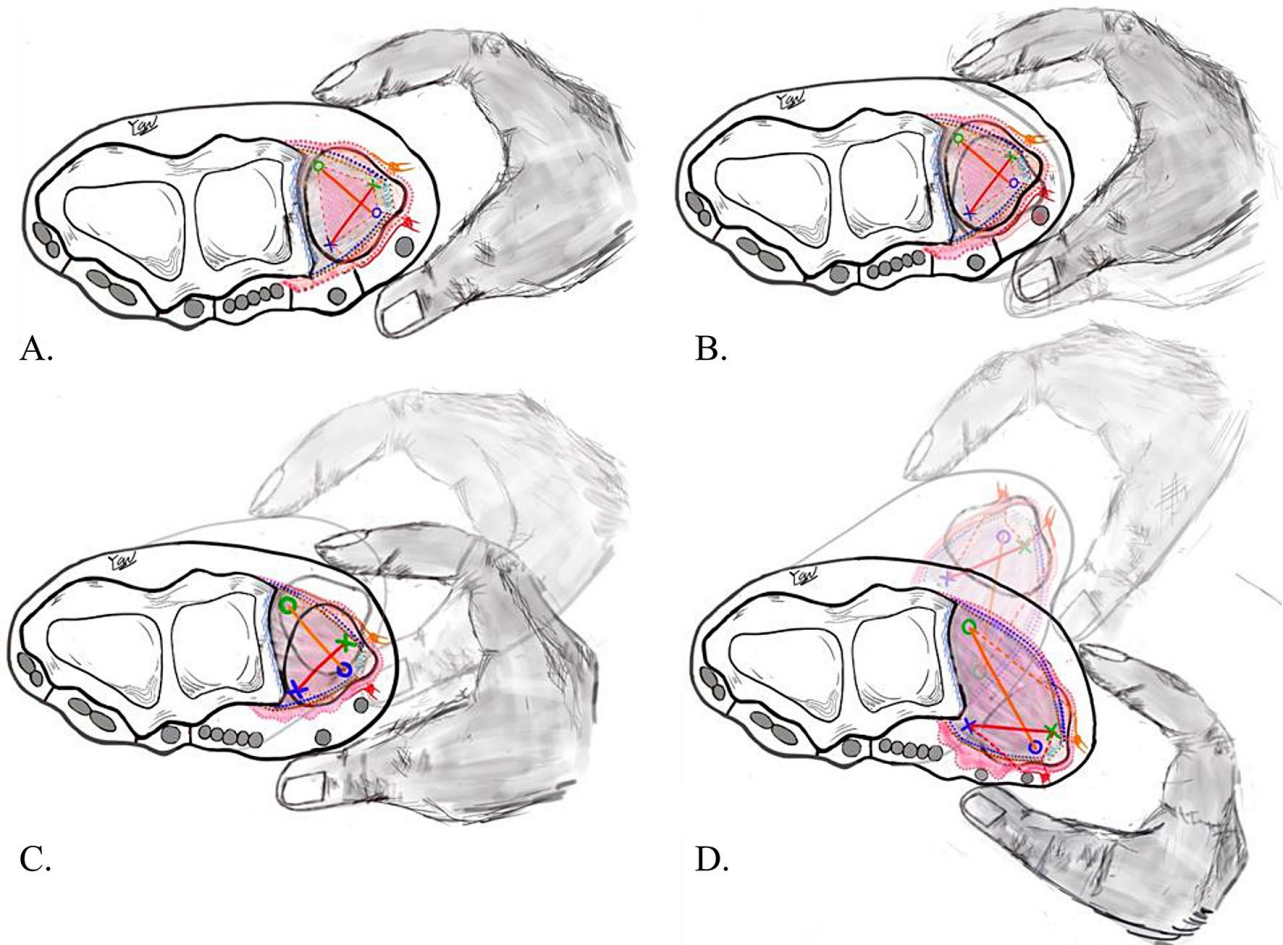
Part II. Intra-operative Ballottement test. **(A)** Grade 1: Normal stability (relative displacement 0%). **(B)** Grade 2: Increase laxity with firm endpoint response to stress (relative displacement 0–25%). **(C)** Grade 3: Increase laxity without firm endpoint response to stress (relative displacement 25–50%). **(D)** Grade 4: Subluxation with passive range of motion (relative displacement >50%)

We employed the intra-operative ballottement test to assess DRUJ stability after completing the “Cross-form” TFCC transcapsular repair, categorizing it into four grades:

- Grade 0: Normal stability (Fig. 4A). In cases where normal stability is detected, the “Cross-form” TFCC transcapsular repair alone is assumed to provide sufficient DRUJ stability.
- Grades 1–3: If there is laxity greater than grade 0 in the intraoperative ballottement test after tightening the strings following TFCC repair, dorsal DRUJ capsular imbrication is performed to stabilize the DRUJ [30].

### Part 3. Dorsal DRUJ capsular imbrication

**Fig. 5:**
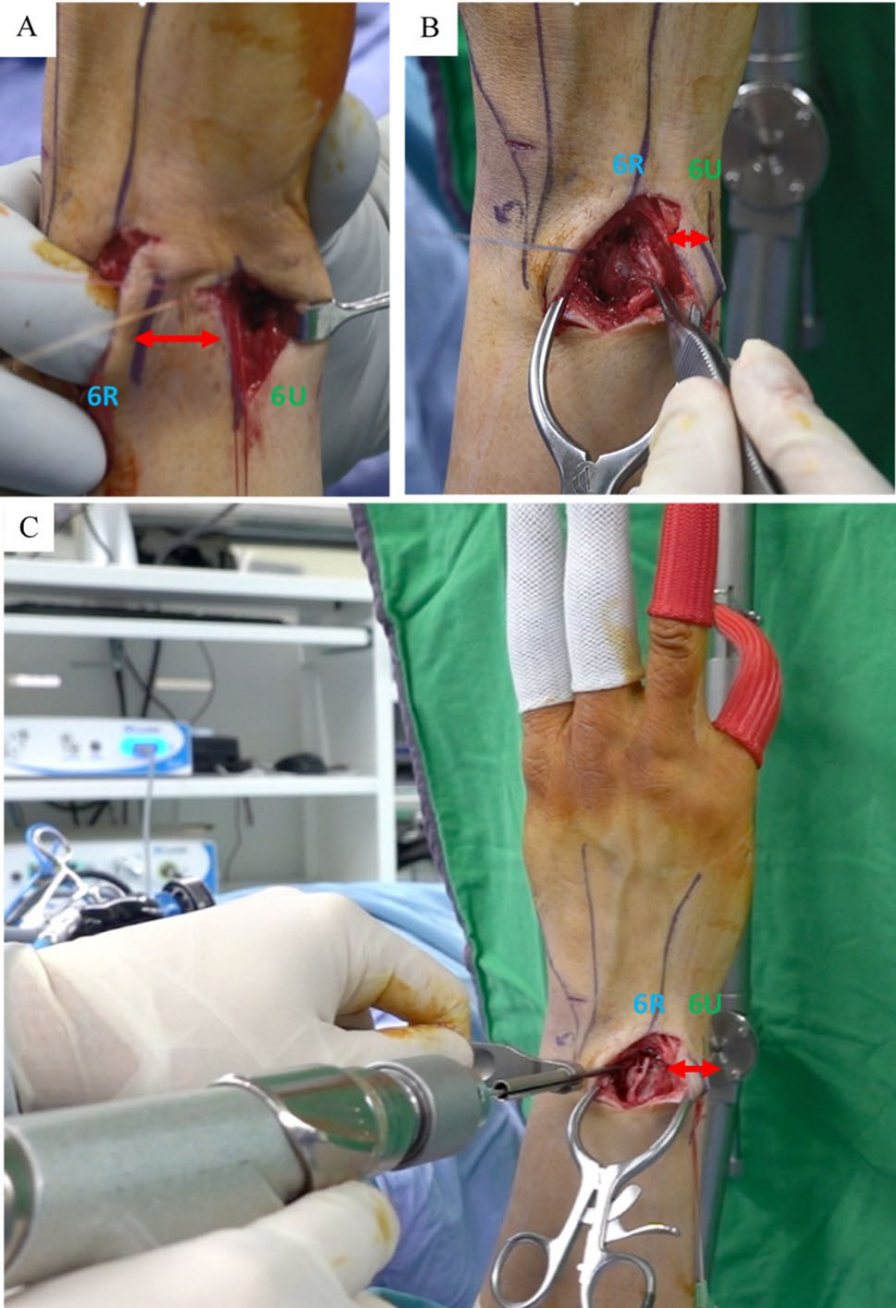
Distance between 6R portal and 6U portal **(A)(B)(C)** Part 1 “Cross-form” TFCC transcapsular repair: 2 cm wound 6U portal; Part 3. Dorsal DRUJ Capsular Imbrication: 4 cm wound extended from 6R portal and incised along EDM; At least 3 cm distance between 6R portal and 6U portal. Legends: Green color: 6U portal (wound for part 1 “Cross-form” TFCC transcapsular repair). Blue color: 6R portal (wound for part 3 dorsal DRUJ Capsular Imbrication). Red color: distance between 6R portal and 6U portal

A 4-cm curved incision, **distance between these two wounds will be at least 3 cm (**Fig. 5**)**, was made along the extensor digiti minimi (EDM) tendon extending proximally to the proximal margin of the DRUJ. Meticulously dissection of subcutaneous tissue was performed, with attention to the dorsal branch of the ulnar nerve. Following the longitudinal incision of the extensor retinaculum, the fourth and fifth extensor compartments were retracted ulnarly and radially, respectively. Subsequently, the dorsal DRUJ capsule was opened and incised longitudinally.

**Fig. 6:**
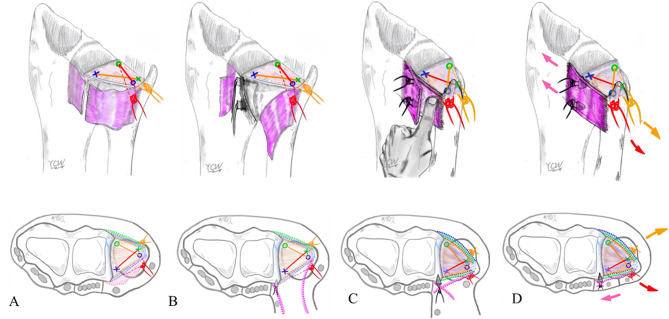
Part III. Dorsal capsular imbrication. **(A)** Chronic DRUJ instability s/p part 1. “Cross-form” TFCC transcapsular repair, the dorsal capsule remains loose. **(B)** Incision of the dorsal capsule into ulnar-based flap and applying two suture anchors over the dorsal cortex near the sigmoid notch. **(C)** Reduction of ulna head with assistant’s thumb in full forearm pronation. **(D)** Operator tightened the knots to maintain the DRUJ’s reduction after restoring the normal alignment of the DRUJ. Legends: TFCC, triangular fibrocartilage complex. DRUJ, distal radioulnar joint

In cases of chronic DRUJ instability, the dorsal capsule often exhibited looseness and weakness due to repetitive dorsal stretching by the ulnar head (Fig. 6A). A rectangular capsule flap, approximately 2 × 2.5 cm^2^ and ulnar-based, was carefully dissected from the dorsal cortex of radius bone, extending from the radial to ulnar direction, and exposing the radius sigmoid notch and ulnar head (Fig. 6B). To enhance the healing potential of DRUJ capsule-to-bone connection, the dorsal cortex of distal radius was decorticated using a rongeur. Two 1.4 all-suture bone anchors (JuggerKnot; Zimmer Biomet, Warsaw, IN) were individually placed radially over the upper and lower borders of the distal radius sigmoid notch (Fig. 6B).

Subsequently, with the elbow flexed at 90º and the forearm in a straightened position with full pronation, the assistant digitally pressed the dorsally displaced ulnar head, lowering it back into the sigmoid notch. The operator then imbricated the detached radius- and ulnar-based capsule flap by tightening sutures from the bone anchors (Fig. 6C). This maneuver stabilized the ulna head in a secured position (Fig. 6D).

The patient was protected with a long-arm cast, with the forearm in a neutral position, for the first 4 weeks postoperatively. After cast removal, passive three-dimensional (3D) wrist motions were initiated with wrist brace protection from 5 to 8 weeks postoperatively. Low-intensity muscle strengthening exercises were introduced from weeks 9–12 postoperatively.

### Postoperative course and follow-up

The patient’s profile, time interval from injury to surgery, and intra-operative and post-operative complications were documented based on the medical charts. The push-off test and ballottement test were employed to evaluate the ulnar-side pain relief and DRUJ stability, respectively. At postoperative intervals of 3, 6, 9, 12, 24, and 36 months, active motion arcs were measured using a goniometer and grip strength was measured with the Jamar Hydraulic Hand Dynamometer (Jamar Technologies/America, Hatfield, PA).

Additionally, patient-reported outcomes, including MMWS, Patient-Rated Wrist Evaluation (PRWE), and Disabilities of the Arm, Shoulder, and Hand (DASH) were used. The proportion of patients meeting the minimal clinically important difference (MCID) of the DASH (MCID: 10–13.5) and PRWE scores (MCID: 14–17) allowed for the quantitative recording of the direct feelings of the patients [31].

### Post hoc power analysis

In our prior comparative research [20], the mean ± standard deviation of wrist range of motion (ROM), with respect to pronation and supination, was found to be 161 ± 13.6º, and 156 ± 12.6º in the “dorsal capsular imbrication” group and the “TFCC repair + dorsal capsular imbrication” group, respectively. Based on a statistical power of 80% and a significance level of 5%, we determined that a minimum of 90 cases for group 1 and 90 cases for group 2 were necessary to ascertain whether a true difference in clinical outcomes existed between both groups.

### Statistical analysis

All data were analyzed using SPSS software (version 20.0; IBM Corp., Armonk, NY). The Shapiro–Wilk test showed that the data were normally distributed; therefore, parametric tests were employed for comparison. Categorical variables were presented as frequency (%). The Chi-squared test was used for parametric statistical analysis of categorical information, and the independent sample T test was employed for parametric analysis of continuous variables. To compare outcome measurements between two groups (DASH score, PRWE score, grip strength, and ROM), the Wilcoxon rank sum test was used. Statistical significance was set at *P* < 0.05.

## Results

**Fig. 7:**
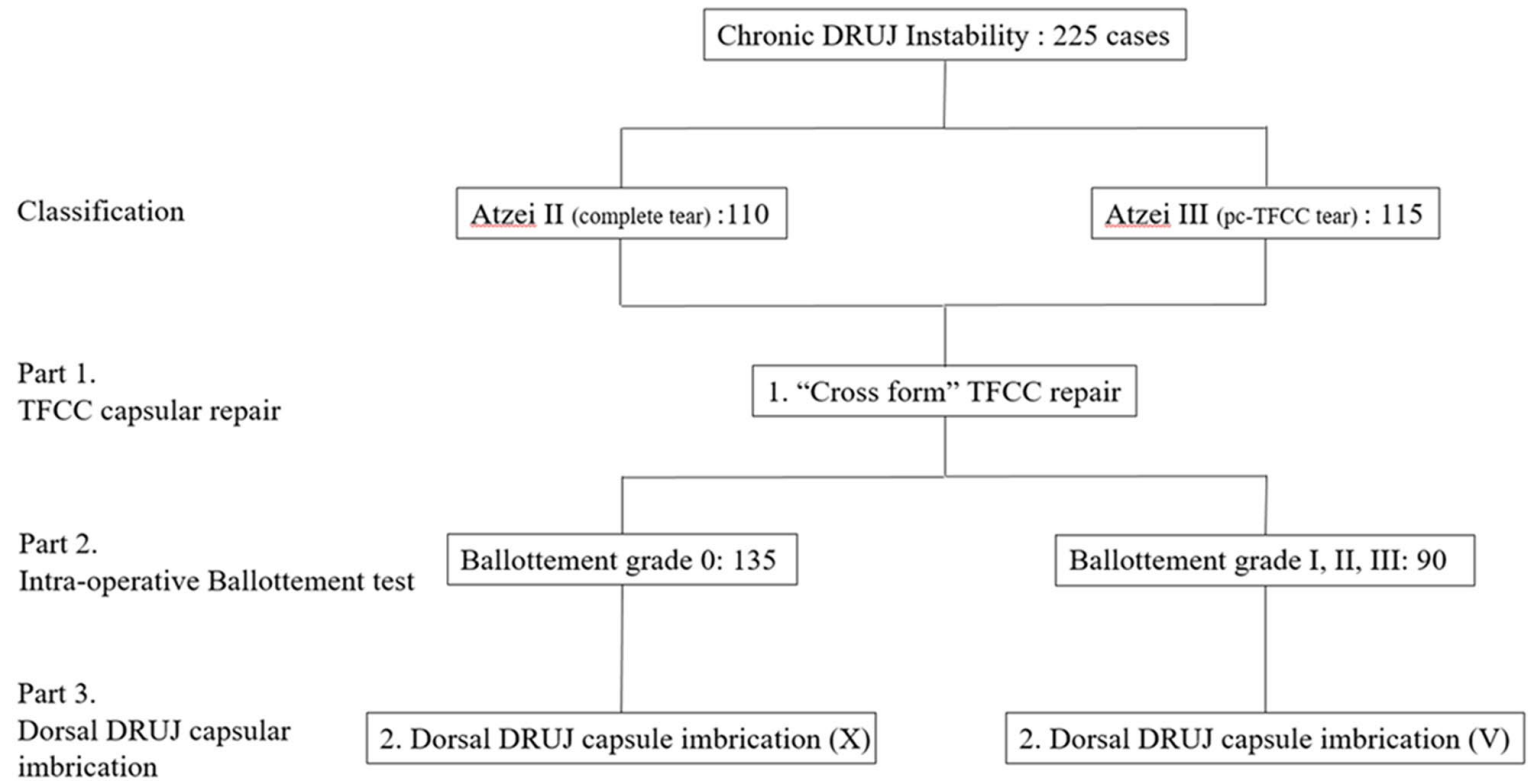
Treatment protocol of patients with chronic DRUJ instability. Legends: TFCC: triangular fibrocartilage complex. DRUJ: distal radioulnar joint

From January 2016 to June 2021, a total of 265 patients underwent surgical treatment for post-traumatic chronic DRUJ instability at our hospital. Among them, those excluded from the study were 29 patients who underwent DRUJ reconstruction due to Atzei class IV or V TFCC tear, 4 with radioulnar joint arthritis, 7 lost to follow-up, and 4 who had prior wrist surgery. Ultimately, a total of 225 patients were included in our final analysis. Among them, 110 had Atzei class II and 115 had Atzei class III TFCC tears, and all underwent arthroscopy-assisted TFCC capsular repair with dorsal DRUJ capsule imbrication (Fig. 7).

This study comprised 130 (57.8%) men and 95 (42.2%) women, with right-sided DRUJ instability occurring in 142 (63%) and left-sided in 83 (36%) cases. The patients’ ages ranged from 22 to 58 years (mean, 41 years). The duration of symptoms before surgery ranged from 6 to 24 months (mean, 12.7 months; range, 6–24 months). The mean follow-up time was 45 months (range: 36–60 months) (Table 1). Table 2 presents the demographic and clinical characteristics of the patients, who were divided into two groups: Group 1, “Cross form” TFCC repair, and Group 2, “Cross form” TFCC repair + DRUJ dorsal capsular imbrication, with no significant difference in each variable category.

**Table 1:** Patients demographic and clinical characteristics

Variable	CR^a^	CR + DCI^b^
Number	135	90
Sex		
Female	55	40
Male	80	50
Hand (R/L)	76/59	66/24
Age (years)	41.5 (25–58)	36.3 (22–55)
Symptoms to surgery (months)	12.2 (6–24)	13.5 (6–24)
Follow-up (months)	47.1 (36–60)	44.7 (36–60)
Atzei classification		
II	82	40
III	53	50

CR^a^: Cross-form repair

CR + DCI^b^: Cross-form repair + Dorsal capsular imbrication

**Table 2:** Cross-form repair group: 135 cases (pre-operative vs. post-operative 3 years)

Variable	Pre-operative	Post-operative	*P* value
Grip strength^a^	50% ± 21%	90.1% ± 5%	< 0.05
Wrist ROM^b^			
Flex-extension	52.3% ± 17%	95.4% ± 5%	< 0.05
Supi-pronation	47.3% ± 22%	92.4% ± 2%	< 0.05
Radial-ulnar deviation	57% ± 18%	90.5% ± 5%	< 0.05
DASH^c^ score	51.6 ± 14.2	9.9 ± 4.2	< 0.05
PRWE^d^: score	40.7 ± 10.3	10.5 ± 5.7	< 0.05
MMWS^e^	50% ± 21%	95.1% ± 5%	< 0.05

Grip strength^a^ (op/non-op) × 100%

Wrist range of motion^b^ (op/non-op) × 100%

DASH^c^: Disabilities of the Arm, Shoulder, and Hand

PRWE^d^: Patient-Rated Wrist Evaluation

MMWS^e^: Modified Mayo Wrist score.

The preoperative and 36-month postoperative scores for DASH, PRWE, grip strength, MMWS, and wrist ROM (flexion-extension + pronation-supination + radial-ulnar arcs) are shown in Table 2 (Group 1, Cross-form repair group) and Table 3 (Group 2, Cross-form repair + Dorsal capsular imbrication group), and all significant differences were identified with P values < 0.05. Additionally, patient-reported outcomes scores showed that 95% (214 in 225) of patients achieved the MCID for DASH scores, and 92% (207 in 225) achieved the MCID for PRWE scores.

**Table 3:** Cross-form repair + dorsal capsular imbrication group: 90 cases (pre-operative vs. post-operative 3 years)

Variable	Pre-operative	Post-operative	*P* value
Grip strength^a^	47% ± 17%	95.1% ± 5%	< 0.05
Wrist ROM^b^			
Flex-extension	51.3% ± 20%	92.4% ± 3%	< 0.05
Supi-pronation	49.2% ± 19%	91.1% ± 3%	< 0.05
Radial-ulnar deviation	56% ± 18%	88.2% ± 4%	< 0.05
DASH^c^ score	50.1 ± 17.1	10.2 ± 4.2	< 0.05
PRWE^d^: score	41.7 ± 11.4	9.5 ± 5.1	< 0.05
MMWS^e^	44% ± 25%	93.7% ± 5%	< 0.05

Grip strength^a^ (op/non-op) × 100%;

Wrist range of motion^b^ (op/non-op) × 100%

DASH^c^: Disabilities of the Arm, Shoulder, and Hand

PRWE^d^: Patient-Rated Wrist Evaluation

MMWS^e^: Modified Mayo Wrist score

Comparison of post-operative results between Group 1 and Group 2 are shown in Table 4. Our findings revealed that in the mid-term (post-operative 1 year to 3 years), the “CR + DCI” group continued to demonstrate superior grip strengths compared with the “CR” group (Table 4). However, no significant difference was observed in all directions of wrist ROM between the two groups at the post-operative 3-year follow-up (Table 4).

**Table 4:** Cross-form repair group vs. cross-form repair + dorsal capsular imbrication group (post-operative 3 years)

Variable	CR^g^	CR + DCI^h^	*P* value
Grip strength^a^	90.1% ± 5%	95.1% ± 5%	< 0.05
Wrist ROM^b^			
Flex-extension	95.4% ± 5%	92.4% ± 3%	> 0.05
Supi-pronation	92.4% ± 2%	91.1% ± 3%	> 0.05
Radial-ulnar deviation	90.5% ± 5%	88.2% ± 4%	> 0.05
DASH^c^ score	9.9 ± 4.2	10.2 ± 4.2	> 0.05
PRWE^d^: score	10.5 ± 5.7	9.5 ± 5.1	> 0.05
MMWS^e^	95.1% ± 5%	93.7% ± 5%	> 0.05
Complications			
**Recurrent instability (%)**	**3.7%**	**1.1%**	**< 0.05**
Transient DSBUN^*f*^ irritation	2.9%	2.2%	> 0.05

Grip strength^a^ (op/non-op) × 100%;

Wrist range of motion^b^ (op/non-op) × 100%

DASH^c^: Disabilities of the Arm, Shoulder, and Hand

PRWE^d^: Patient-Rated Wrist Evaluation

MMWS^e^: Modified Mayo Wrist score

DSBUN^f^: Dorsal sensory branch of the ulnar nerve

CR^g^: Cross-form repair

CR + DCI^h^: Cross-form repair + Dorsal capsular imbrication

Post-operative complications included that recurrent DRUJ instability, which occurred in 3.7% (5/135) and 1.1% (1/90) in Group 1 and Group 2, respectively, with a significant difference between the two groups. Also, the transient dorsal sensory branch of the ulnar nerve (DSBUN) irritation was noted with 2.9% (4/135) and 2.2% (2/99) in Group 1 and Group 2, separately with no significant difference between the two groups (Table 4).

Notably, a total of 95% (214/225) of patients achieved pain relief in the push-off test, 97.3% (219/225) regained DRUJ stability in the ballottement test, and only 1.8% (4/225) required re-operation due to DRUJ osteoarthritis changes after 3 years postoperatively. Moreover, patient-reported outcomes indicated that 91% and 92% of patients achieved the MCID in the DASH and PRWE scores, respectively [31].

## Discussion

The primary finding of our study is that incorporating DCI in TFCC repair for patients with a positive intraoperative ballottement led to a very low post-operative instability rate. Compared with patients who underwent TFCC repair alone (due to intraoperative negative ballottement test), those who received DCI as an augmentation demonstrated significantly higher grip strength, short-term decreased wrist ROM, and long-term comparative ROM.

According to the Atzei classification, TFCC fovea tear (class II, III) required foveal TFCC repair [4, 32]. The neglected TFCC fovea tear might contribute to chronic DRUJ instability [5] resulting in decreased grip strength or limited wrist ROM [33]. Despite the favorable outcomes reported for “transosseous repair [6–8]” “fovea repair with suture anchors [4, 9, 10]”, re-operation rates have been documented in the range of 6.7–30% [8, 34–37]. Discrepancies in clinical results and reduced efficacy of fovea repair may be attributed to (1) the poor quality or irreparable remnants of TFCC fovea tears that cannot stabilize DRUJ, (2) insufficient coverage area for sutures or knots, increasing the risk of TFCC cut-through during knot tying, and (3) inadequate foveal debridement or improper positioning of bony tunnels, leading to limited bone-to-ligament regeneration capacity.

Recent studies comparing DRUJ stability after capsular repair and transosseous repair have produced varying results: Ruch et al. [38] demonstrated no significant difference, while Johnson et al [39]. indicated greater stability with transosseous repair. However, the critical factor for successful TFCC repair lies in the healing potential of the contact surface, w6hich is notably poor in ligament-to-bone repair (fovea repair): 1. Ulna fovea has a “band shaped”-like footprint [40], whereas “suture anchor repair” and “transosseous tunnel repair” only provide a point contact area between the TFCC remnant and the ulna fovea; 2. “ Enthesis” refers to the insertion site of a tendon, ligament or joint capsule into bone [41]. Fovea repair, “transooseous repair” or “suture anchor repair,” requires the reattachment of TFCC remnant parts into the ulna fovea. Few vessels penetrate the enthesis due to a calcified barrier [42]. In contrast, capsular repair may be more effective in enhancing the healing potential of the TFCC through ligament-to-capsule repair compared to [43] ligament-to-bone repair. However, a comprehensive review involving 825 cases across 30 studies revealed post-operative distal radioulnar joint (DRUJ) instability rates of 12.1% for capsular repair and 10.1% for fovea transosseous repair. Regarding re-operation rates, they were 7.9% for capsular repair and 5.5% for fovea transosseous repair [16]. These results indicate that intraoperative instability of the DRUJ can be a concern in both primary methods of TFCC repair. Therefore, employing an intraoperative DRUJ stability test could be essential for identifying potential postoperative instability and the failure of TFCC repair. Augmentation with DCI can help prevent postoperative DRUJ instability and the need for subsequent reoperation.

The intra-operative ballottement test is a simple method for evaluating DRUJ stability after arthroscopic TFCC repair. A positive result suggests that the strength of the repaired TFCC alone may be insufficient to maintain DRUJ stability. DCI can be employed as a supplementary method to enhance DRUJ stability. Using DCI as a sole treatment for patients with DRUJ instability has been successful in restoring DRUJ stability in 97.8% of cases, with 93.6% of patients experiencing pain relief through this approach [17–25]. In a long-term study spanning 10 years, it was observed that DCI effectively restored wrist function to levels comparable to the contralateral hand. DCI can also function as a secondary stabilizer, following a similar bridging concept to that of the internal brace used in anterior talofibular ligament [44] or knee medial collateral ligament repair [45]. When combined with the suture tap and bone anchors, it can reinforce ligament strength and prevent injury recurrence during the rehabilitation process [44]. Similarly, DCI can restore intact DRUJ kinematics and radioulnar ligament reconstructions in chronic DRUJ instability [46]. In the present study, recurrent DRUJ instability was found to be significantly lower in patients with the augmentation of DCI, compared to 3.7% and 1.1% in “CR” group 1 and “CR + DCI” group 2, with a significant difference. Thus, we believe that DCI could be an effective method for addressing intraoperative DRUJ instability following TFCC capsular repair.

In this treatment protocol, we aim to outline the procedures necessary to restore the integrity of TFCC and DRUJ capsules: (1) “TFCC capsular repair” combines the benefits of the inside-out and outside-in techniques, reducing the cut-through rate, purchasing the wide contact area between the ulna fovea and adhering TFCC remnant part with the surrounding tissue to reinforce the DRUJ stability. The crux of transcapsular repair is the ligament-to-soft tissue healing process. Therefore, non-absorbable suture 2 − 0 ethibond was selected to provide reliable tension support. (2) “intra-operative Ballottement test” checked the integrity of the DRUJ stability, grade 0 indicates that “TFCC transcapsular repair” was sufficient to maintain DRUJ stability, while grade I, II or III suggests that DRUJ laxity or subluxation existed after transcapsular repair, and the subsequent augmentation for DRUJ stability was needed, (3) “Dorsal DRUJ capsular imbrication” tightening the redundant laxity of dorsal DRUJ capsule, reducing the subluxation of ulna head and reattaching the DRUL to the tightened DRUJ capsule under wrist full-pronation position. Tension of the imbricated capsule can be optimized to stabilize DRUJ with the utilization of two suture anchors over the dorsal cortex of the radius sigmoid notch. Our results indicated a slightly higher rate of postoperative distal radioulnar joint (DRUJ) instability in Group 1, which underwent only TFCC capsular repair, compared to Group 2, which received both TFCC capsular repair and dorsal DRUJ capsular imbrication. This implies that late DRUJ instability may manifest in patients who initially tested negative in the intraoperative ballottement test but only underwent TFCC repair. It also implies that DCI is a reliable procedure to build up the DRUJ stability.

A major concern about our methods was that there was no wrist stiffness found in the “CR + DCI group” compared with “CR” group” in the post-operative 3 years following. Additionally, the grip strength of “CR + DCI group” showed significantly better than that of “CR” group”. Another issue to consider is that the transient neuropathy occurred in 2.7% (6/225) patients due to dorsal sensory branch of ulnar nerve (DSBUN) needed to be retracted and protected during tying knot process; however, sensory irritation will subside within 2 weeks postoperatively.

### Limitations

This study has few limitations. First, it focused solely on surgical outcomes and functional measures, lacking postoperative axial MRI to verify the repositioned DRUJ. Second, being a retrospective comparative study with post-operative 3 years follow-up, a longer-term investigation is needed to validate the observed clinical outcomes. Third, the intraoperative ballottement test employed in this study remains subjective. Future studies should consider standardizing pull strength and translation distance measurements to enhance the accuracy of identifying subtle cases of DRUJ instability following TFCC repair. Finally, we did not include a control group comprising patients with persistent instability after TFCC repair who did not receive additional treatment to enhance DRUJ stability. However, establishing such a control group presented ethical and clinical challenges, as leaving untreated cases of persistent DRUJ instability was not considered feasible.

## Conclusions

Our findings revealed that in chronic cases of DRUJ instability with ulna fovea tear, “Cross form” TFCC repair may be employed to restore DRUJ stability. Specifically, if the intra-operative ballottement test indicates residual DRUJ instability following TFCC capsular repair, “Dorsal capsular imbrication” can be applied to augment DRUJ stability. This procedural protocol serves as a viable treatment option for patients experiencing chronic DRUJ instability.

## Data Availability

The datasets generated and analysed during the current study are not publicly available as the participants’ informed consent only permits data usage within this study and prohibits the sharing of their personal data with the public, but are available from the corresponding author on reasonable request.
